# Congenital Laryngeal Cyst as a Rare Cause of Stridor in Infants: Two Case Reports

**DOI:** 10.7759/cureus.33867

**Published:** 2023-01-17

**Authors:** Samuel Chu, Farhana A, Farid R, Saraiza Abu Bakar

**Affiliations:** 1 Otolaryngology, Hospital Shah Alam, Shah Alam, MYS; 2 Otolaryngology, Hospital Serdang, Kajang, MYS

**Keywords:** stridor, congenital laryngeal cyst, paediatric ent, laryngeal lesions, paediatric otolaryngology, otolaryngology case report, otolaryngology

## Abstract

Congenital laryngeal cysts are a rare cause of stridor in infants. These cysts may have serious clinical implications if not promptly recognized. A small laryngeal cyst may remain asymptomatic. However, if large, it can block the laryngeal inlet leading to acute airway obstruction, which is potentially life-threatening. In pediatric patients presenting with respiratory distress, prompt diagnosis and surgical management are crucial to avoid infant morbidity and mortality. We describe two cases of laryngeal vallecular cysts in infants and their management in our clinical practice.

## Introduction

Stridor in an infant is related to several pathologies of the airway. When accompanied by respiratory distress in a neonate, it must be addressed urgently. More common pathology includes laryngomalacia, tracheomalacia, vocal cord paralysis, and subglottic stenosis, among others. Although rare, congenital laryngeal cysts are one of the causes of stridor in an infant [1]. Congenital laryngeal cysts can be divided into two types: ductal and saccular. Ductal cysts arise as a result of obstruction of laryngeal epithelial mucous glands; they make up 75% of all congenital laryngeal cysts. Saccular cysts are formed when there is mucus retention in the laryngeal saccule due to atresia or obstruction of the laryngeal ventricle orifice [2]. Diagnosis of congenital laryngeal cyst can be made by laryngoscopy and can be managed by endoscopic deroofing excision. In this report, we detail two cases of neonatal stridor secondary to congenital laryngeal cysts.

## Case presentation

Case 1

**Figure 1:**
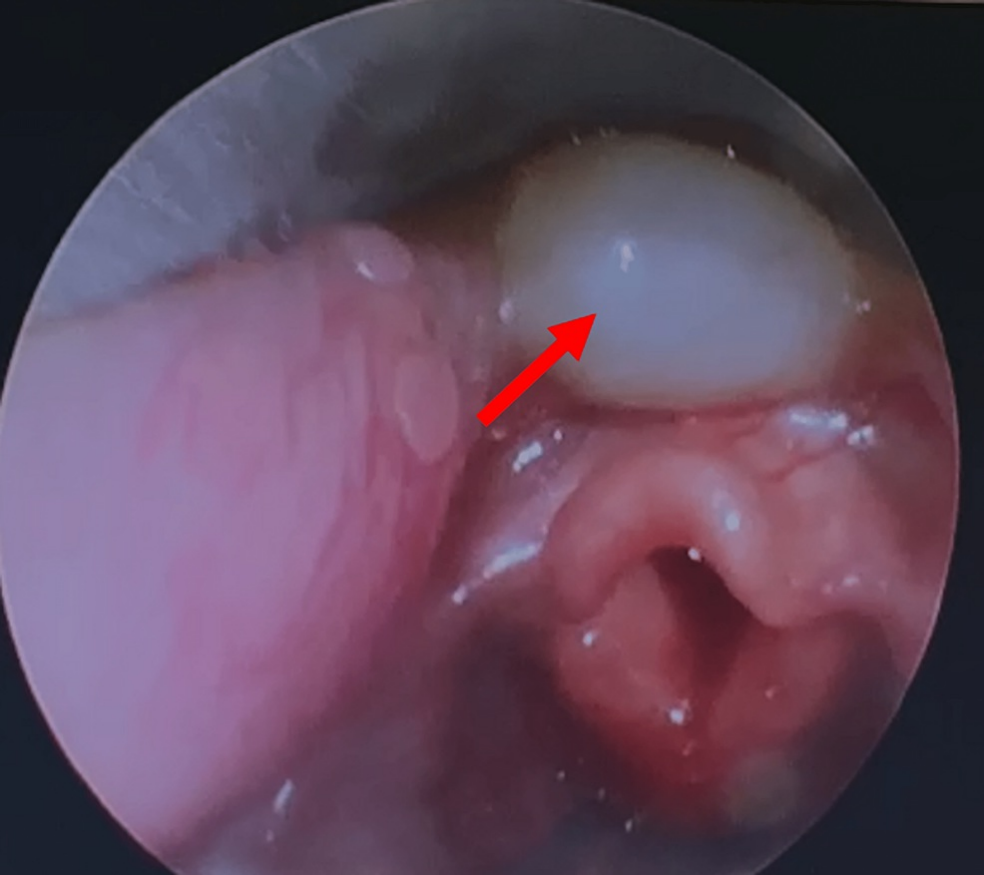
Congenital laryngeal cyst with features of laryngomalacia (omega-shaped epiglottis with tight aryepiglottic folds seen).

An eight-day-old baby girl was referred to the otorhinolaryngology team for stridor. She was born prematurely at 34 weeks and three days via spontaneous vaginal delivery. Her Apgar score was 4 out of 10 at birth; hence, she was intubated at four minutes of life. She was subsequently extubated on day two of life; however, she persistently required non-invasive oxygen supplementation. She was noted to have noisy breathing. Physical examination found soft stridor with minimal substernal and subcostal recession. She also had features of soft dysmorphism, microcephaly, flat nasal bridge, and hypertelorism. Bedside flexible laryngoscopy was done on her second week of life, revealing a floppy epiglottis, redundant arytenoid mucosa, and secretions at the subglottic region with the presence of a small laryngeal cyst at the valleculae. The baby underwent direct laryngoscopy and examination under general anesthesia, which revealed laryngomalacia (omega-shaped epiglottis with tight aryepiglottic folds) and subglottic stenosis with synchronous vallecular cyst (Figure 1). A Hopkins telescope (zero degrees) was introduced transorally to facilitate visualization of the vallecular cyst endoscopically. Marsupialization of the cyst was performed with microlaryngeal instruments. Hemostasis was secured with the application of topical adrenaline and diathermy. During subsequent follow-up (six months of life), the baby was well and a flexible laryngoscopy revealed a normal larynx.

Case 2

**Figure 2:**
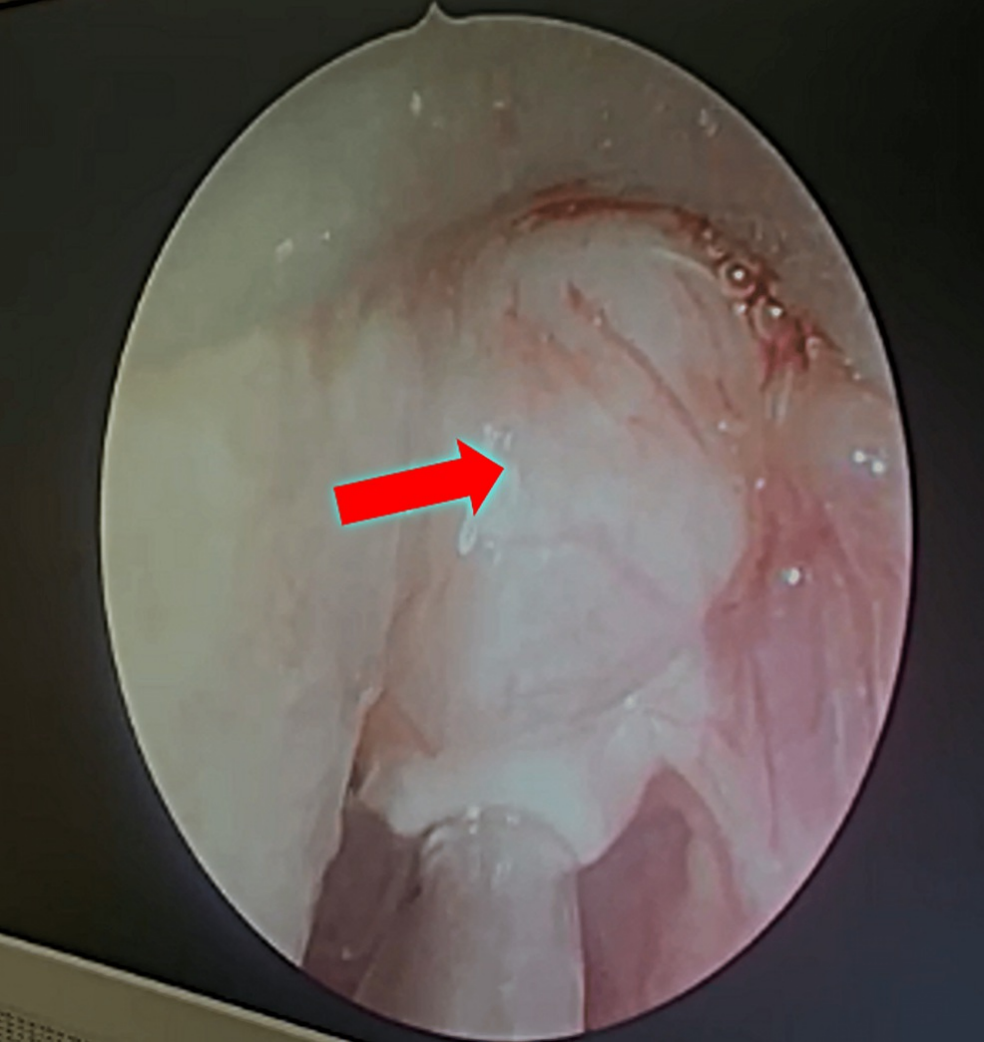
Congenital laryngeal cyst with features of laryngomalacia (omega-shaped epiglottis with tight aryepiglottic fold).

A two-month-old baby boy was delivered with a full Apgar score. He had neonatal jaundice requiring low-intensity phototherapy and was subsequently discharged to home. His mother noticed that he had noisy breathing since the second week of life. The condition worsened when the child was active. Furthermore, the child started developing difficulty with feeding. The infant was admitted and referred to the otorhinolaryngology team. Physical examination noted soft intermittent inspiratory stridor with subcostal and intercostal recession. Oxygen saturation was 100% under continuous positive airway pressure (CPAP). Bedside flexible laryngoscopy showed features of laryngomalacia, tubular epiglottis, tight aryepiglottic fold, and floppy arytenoids. There was a pooling of secretions at the pyriform fossae. The child underwent direct laryngoscopy under general anesthesia, which revealed a cystic mass over the valleculae (Figure 2). Similarly, a Hopkins telescope (zero degrees) was introduced transorally to facilitate visualization of the vallecular cyst endoscopically. Deroofing of the laryngeal cyst and supraglottoplasty was performed in the same setting with microlaryngeal instruments. Repeated flexible laryngoscopy later showed a normal larynx with no recurrence of the cyst.

## Discussion

Congenital stridor can be caused by several conditions. These conditions can be broadly divided into supraglottic, glottic, and subglottic conditions. Laryngomalacia is the single most common congenital laryngeal abnormality causing stridor. This is followed by subglottic stenosis, vocal cord paralysis, and laryngeal webs [3]. Laryngeal cyst remains a very rare cause of neonatal respiratory obstruction. The incidence of congenital laryngeal cysts is approximated to be 1.8 in 100,000 newborns [4]. However, this may be an underestimation, as some small cysts do not produce symptoms and are often undetected.

The clinical presentation of laryngeal cysts varies according to site and size [2]. A larger cyst often presents with respiratory distress at birth. However, a small cyst may be asymptomatic or present with mild respiratory distress. In both of our cases, the diagnosis of laryngeal cysts was not made at birth. However, mild and discrete features of respiratory distress along with a persistent requirement for oxygen supplementation prompted an otolaryngology consult. Bedside flexible nasal laryngoscopy suggested the diagnosis of a congenital laryngeal cyst and direct laryngoscopy in the operating room under general anesthesia confirmed the diagnosis. Hence, laryngomalacia, although common, is not always the only cause of stridor in newborns. Endoscopic airway evaluation is essential as part of the clinical assessment of a child presenting with stridor [5].

Minimally invasive surgery and cyst removal is the treatment choice for congenital laryngeal cysts. However, there are several surgical modalities for the treatment of congenital cysts. They include endoscopic excision, deroofing, and needle aspiration. This is done after securing the airway with endotracheal intubation, and tracheostomy is performed in some cases where endotracheal intubation is not possible [6]. Needle aspiration is considered to be inadequate as a high risk of recurrence of cysts is widely reported. Endoscopic deroofing or complete excision with a carbon dioxide (CO2) laser or instrument is recommended.

The surgery was performed in the same setting as confirmation of diagnosis with direct laryngoscopy under general anesthesia. A vallecular cyst may often be approached via the transoral route, and an external approach is required only in rare cases due to large and recurrent lesions [7]. In both of the cases described, a simple, direct, and transoral approach for managing vallecular cysts was described. This approach can be considered to be a safe approach for infants with laryngeal vallecular cysts and the recurrence rate using this approach is low [8]. Chen et al. found no recurrence in seven patients who underwent surgery for the treatment of vallecular cysts with a similar approach [8]. In the above-described cases, no recurrence was observed during the follow-up period, which ranged from 10 months to two years.

## Conclusions

Both cases described how a benign swelling can potentially lead to potentially life-threatening airway compromise. Although rare, congenital laryngeal cysts should be considered as part of our differential diagnosis when attending to an infant with stridor.

Laryngoscopy should be included as part of the assessment when approaching a child with stridor. Prompt diagnosis, if made, can lead to early minimally invasive surgical procedures, which are simple, safe, and effective to avoid potentially life-threatening airway obstruction.
